# 

**Published:** 1915-07

**Authors:** 


**CONTENTS, JULY, 1915—VOLUME XXXI, NO. 1.**

ORIGINAL ARTICLES.

Carcinoma of the Colon; Its Pathology and Symptoms. By Claude C. Cody, M. A., M. D. .... 1

The X-Ray in the Treatment of Malignancy. By B. T. Van Zant, M. D., Houston, Texas .... 8

EUGENICS DEPARTMENT.

Edited by Malone Duggan, San Antonio.

Heredity and Cancer .... 12

A Warning .... 12

Higher Education and Race Suicide .... 13

The Evolution of Character .... 14

Legal Limitation of Family Limitation. By Dr. G. Henri Bogart, Paris, Ill .... 17

EDITORIAL DEPARTMENT.

The Waste of Life from Cancer. By John T. Moore, A. M., M. D., Houston Texas .... 23

"Articles of Faith" Concerning Cancer—A Platform Upon Which to Unite in the Campaign of Education .... 29

Words of Encouragement .... 31

Items of General Interest .... 33

Personal News Items .... 34

Deaths .... 37

Book Reviews .... 38

Helpful Hints for the Busy Doctor .... 40

